# Availability of essential medicines in selected public, primary and secondary health care institutions of a rural Sri Lankan district: a spot survey

**DOI:** 10.1186/s12913-016-1969-2

**Published:** 2017-01-05

**Authors:** Devarajan Rathish, Indika Premarathna, Thiwanka Jayathilake, Chathurika Kandegedara, Kalani Punchihewa, Lakmali Ananda, Thejani Bandara, Channa Jayasumana, Sisira Siribaddana

**Affiliations:** 1 Department of Pharmacology, Faculty of Medicine and Allied Sciences, Rajarata University of Sri Lanka, Saliyapura, Sri Lanka; 2 Department of Medicine, Faculty of Medicine and Allied Sciences, Rajarata University of Sri Lanka, Saliyapura, Sri Lanka

**Keywords:** Availability, Essential medicine, Rural sector, Sri Lanka, Primary care, Secondary care

## Abstract

**Background:**

Assessment of the availability of essential medicines, in rural areas of countries with free state health care system, is scarce. Dependence on essential medicines among the population in rural sector is considered to be high. Assessing the availability of essential medicines in selected state owned primary and secondary health care institutions of a rural district will help to identify areas where improvement is needed.

**Methods:**

A descriptive cross sectional study, covering selected five primary and one secondary care institutions of a rural Sri Lankan district, was conducted. The national list of essential medicines, Sri Lanka was used as the check list and the guidelines of the WHO–Health Action International were adapted.

**Results:**

The secondary care institution recorded an overall availability of 71%, whereas the average overall availability of the primary care institutions was 56%. Central dispensaries recorded the lowest availability. Lack of availability of medicines needed for the management of chronic kidney disease, snake bite and poisoning was noted.

**Conclusions:**

Availability of essential medicines in most of the primary and the secondary care institutions were fairly high. Deficiency in medicines needed for the management of emergencies was noted. A need based annual estimate of medicines based on an essential medicine list is suggested.

**Electronic supplementary material:**

The online version of this article (doi:10.1186/s12913-016-1969-2) contains supplementary material, which is available to authorized users.

## Background

Access to medicine is an universal human right, and availability of medicine is a worldwide problem. According to the World Health Organization (WHO), “*Essential medicines are those that satisfy the priority health care needs of the population. Essential medicines are intended to be available within the context of functioning health systems at all times in adequate amounts, in the appropriate dosage forms, with assured quality and adequate information, and at a price the individual and the community can afford*” [1].

Essential medicines are not available to 33% of world population and 50% of people in poorest countries of Africa and Asia [2]. In 2008 a study carried out in 36 developing and middle-income countries reported 29–54% availability of generic medicines in public sector, where Africa recorded the lowest and America the highest [3]. The availability of 15 generic medicines ranged from 10% in Yemen to 79% in Mongolia. All regions showed a lower mean availability in the public sector in comparison to the private sector. In another study availability of essential medicines for chronic diseases was less than 7.5% in four low and middle income countries but was 28% in Sri Lanka (SL) [4]. Generic medicines were available and affordable according to a survey carried out over 6 year period in retail pharmacies (semi-government and private) of SL [5]. The 2013 SL national survey showed a “fairly high” (50–80%) availability of selected essential medicines for non-communicable diseases (NCD) in both the private and public sectors [6]. However, another SL study revealed that the availability of key essential medicines for children was “low” (30–49%) in public hospitals [7].

SL’s health indicators are comparable with developed countries of Asia and in 2013 SL’s total expenditure on health was 3.2% of the gross domestic product [8, 9]. Its allopathic health system consist a universal free (non-fee levying) government sector and a fee levying private sector. There are four levels of health care institutions in the country. The primary care institutions are district hospitals (DH), peripheral units (PU), rural hospitals (RH) and central dispensaries (CD). District general hospitals and base hospitals (BH) are secondary care institutions [10].

Previous national surveys have assessed the availability of essential medicines in SL. Published national surveys have de-identified locations in the surveys. Hence we are unable to comment on the urban–rural differences in availability of the essential medicines [5–7]. An abstract published in 2015 revealed Anuradhapura, a rural district of SL, as the district with highest availability (78%) for drugs used in NCD [11]. To our knowledge studies focusing on availability of drugs in rural districts of SL are scarce. The aim of our study was to assess the availability of essential medicines in selected public health care institutions of a district in rural SL.

## Methods

A descriptive cross sectional study was conducted during the first week of August 2016.

### Study setting

The study was conducted in Anuradhapura district of SL where 95% of households are rural [12]. Agriculture is the main employment for 55% of the population [13]. Unemployment rate is 3.1% [13]. The mean monthly household income of the district is 241 USD, compared to 312 USD for the country [14]. These reasons make the free public health care service as the first choice of the people.

One institution each from the following levels of health care were selected conveniently and visited once for data collection: base hospital, district hospital, peripheral unit, rural hospital and two central dispensaries. The second central dispensary was selected according to WHO–Health Action International (HAI) guidelines. It recommends to survey another institution of the same level if the availability of essential medicine is less than 50% at the first surveyed institution [15]. Identities of all the above institutions were kept confidential.

### Instrument

Availability of essential medicines was checked using the national list of essential medicines, SL [16]. The first edition was published in 1958 and the fifth in 2014. Clinical needs, disease prevalence, evidence of efficacy, safety and comparative cost-effectiveness are considered by the selection committee in selecting the essential medicines. The list has 29 major categories of drugs. Out of which only 28 were relevant to the primary and secondary care institutions. Under each category there is a core list and in some a complementary list. The core list includes “a list of minimum medicine needs for a basic healthcare system listing the most efficacious, safe and cost‐effective medicines for priority conditions” [16]. The complementary list includes “essential medicines for priority diseases, for which specialized diagnostic or monitoring facilities, and/or specialist medical care, and/or specialist training are needed” [16]. The drug information includes the international non-proprietary name of the active moiety, dosage forms, strengths and the level of care at which the drug should be available E.g. Paracetamol tablet: 500 mg (primary, secondary, tertiary and specialist hospitals). This list closely follows the WHO model list of essential medicines [17] with modifications according to the needs of SL.

### Data collection, analysis and description of data

Permission was obtained from the regional director of health services and the relevant heads of the institutions. Availability of essential medicines was checked from the drug stores and the pharmacies of the institutions, using the national list of essential medicines, SL by trained MBBS qualified doctors. According to the WHO–HAI method the specific dose and form was surveyed for availability [15]. As with previous studies the availability of the selected medicine was considered regardless of whether it is innovator, generic or branded generic [4, 6]. A medicine was considered as available only if the data collectors physically saw it [15], drug not being expired and being suitable for use. Data was analyzed using Microsoft excel. Descriptive statistics were used to describe data. Drug availability was described according to earlier national and international surveys; <30%: very low, 30–49%: low, 50–80%: fairly high and >80%: high [6, 18]. Each dosage form of a particular medicine was considered as separate items. Medicines available in several core or complementary lists of the national list of essential medicines are counted only once [16].

### Deviations in methodology from the WHO–HAI recommendations

According to our objective we plan to find out the availability of essential medicines in selected public health care institutions of a rural SL district using the national list of essential medicines, SL. Therefore we differed in the following steps:

- Only availability of drugs was checked and data on pricing was not collected as medicines are dispensed free of charge in state sector [6].
- Only institutions of public primary and secondary care were selected as our objective was to find the availability of essential medicines only in these institutions.
- All drugs included in the national list of essential medicines, SL were checked. We did not restrict the survey to 50 selected medicines as recommended by the WHO-HAI.
- Institutions were selected conveniently rather than randomly. Essential medicines are supposed to be “available within the context of functioning health systems at all times”. The aim of the study was to assess the availability of essential medicines in selected public health care institutions of a district in rural Sri Lanka. We do not intend to generalize the data but would only want to highlight the deficiencies in the availability of essential medicines in those selected institutions.

## Results

Numbers for inward, deliveries and out patients for each of the selected institution for the year 2015 were retrieved from the regional director of health service office (Table 1). Availability of essential medicines in BH, DH, PU, RH and CD-1 were 71, 81, 64, 58 and 32% respectively. As the total availability at CD-1 was less than 50%, second central dispensary (CD-2) was surveyed as planned. Availability of essential medicines in CD-2 was also less than 50% (47%). Availability of individual essential medicines is given as an Additional file 1.

**Table 1:** Number of patients for the year 2015 in the selected institutions of Anuradhapura Tables

Institution	Inward	Outpatient	Deliveries
BH	35,207	238,235	3,219
DH	10,548	170,743	95
PU	4,191	78,714	52
RH	5,352	65,603	40
CD-1	NA	15,448	NA
CD-2	NA	18,858	NA

*BH* Base Hospital, *DH* District Hospital, *PU* Peripheral Unit, *RH* Rural Hospital, *CD* Central Dispensary

### Primary care institutions

The highest availability of essential medicine was seen in DH (81%), a primary care institution, the next best was BH (71%), a secondary care institution. Availability was “fairly high” in PU and RH, whereas it was “low” in both the CDs.

DH showed 100% availability in analgesics, anti-allergics, diuretics, immunologicals, genitourinary medicines, psycho-therapeutic medicines, respiratory medicines, vitamins & minerals and neonatal care medicines. However it recorded “low” or “very low” availability in medicines affecting the blood (33%), diagnostic agents (0%) and ophthalmological preparations (33%) (Table 2).

**Table 2:** Availability of essential medicines (by major category) Anuradhapura, 2016

No	Major categories of essential medicines	Need to be present at PCI	Present at each PCI (%)					Average for PCI (%)	Need to be present at SCI	Present at SCI (%)
			DH	PU	RH	CD-1	CD-2			
1	Anaesthetics	4	3 (75)	3 (75)	2 (50)	1 (25)	2 (50)	2.2 (55)	17	17 (100)
2	Analgesics, antipyretics, non-steroidal anti-inflammatory medicines, medicines used to treat gout and disease modifying agents in rheumatoid disorders	6	6 (100)	5 (83)	4 (67)	3 (50)	4 (67)	4.4 (73)	12	8 (67)
3	Anti-allergics and medicines used in anaphylaxis	8	8 (100)	7 (88)	7 (88)	7 (88)	6 (75)	7 (88)	8	8 (100)
4	Antidotes and other substance used in poisonings	6	3 (50)	3 (50)	2 (33)	1 (17)	0	1.8 (30)	9	8 (89)
5	Anticonvulsants/anti-epileptics	11	9 (82)	5 (46)	6 (55)	0	0	4 (36)	12	6 (50)
6	Anti-infective medicines	24	20 (83)	19 (79)	16 (67)	11 (46)	17 (71)	16.6 (69)	47	32 (68)
7	Anti-migraine medicines	7	6 (86)	5 (71)	4 (57)	5 (71)	3 (43)	4.6 (66)	7	6 (86)
8	Anti-neoplastic, immunosuppressive and medicines used in palliative care	N/A	N/A	N/A	N/A	N/A	N/A	N/A	5	3 (60)
9	Anti-parkinsonism medicines	N/A	N/A	N/A	N/A	N/A	N/A	N/A	4	1 (25)
10	Medicines affecting the blood	6	2 (33)	2 (33)	2 (33)	1 (17)	2 (33)	1.8 (30)	8	4 (50)
11	Blood products and plasma substitutes	N/A	N/A	N/A	N/A	N/A	N/A	N/A	1	1 (100)
12	Cardiovascular medicines	26	24 (92)	21 (81)	21 (81)	9 (35)	16 (62)	18.2 (70)	27	24 (89)
13	Dermatological medicines (topical)	18	14 (78)	8 (44)	6 (33)	4 (22)	5 (28)	7.4 (41)	24	8 (33)
14	Diagnostic agents	1	0	0	0	0	0	0 (0)	1	0 (0)
15	Disinfectants and antiseptics	6	5 (83)	3 (50)	4 (67)	2 (33)	3 (50)	3.4 (57)	6	6 (100)
16	Diuretics	4	4 (100)	4 (100)	4 (100)	1 (25)	3 (75)	3.2 (80)	5	5 (100)
17	Gastrointestinal medicines	10	8 (80)	8 (80)	9 (90)	5 (50)	5 (50)	7 (70)	15	12 (80)
18	Hormones, other endocrine medicines and contraceptives	9	8 (89)	7 (78)	7 (78)	4 (44)	6 (67)	6.4 (71)	11	8 (73)
19	Immunologicals	1	1 (100)	0	0	0	0	0.2 (20)	3	3 (100)
20	Muscle relaxant (Peripherally acting) and cholinesterase inhibitors	N/A	N/A	N/A	N/A	N/A	N/A	N/A	2	2 (100)
21	Ophthalmological preparations	3	1 (33)	0	1 (33)	1 (33)	1 (33)	0.8 (27)	3	0 (0)
22	Medicines acting on the genitourinary tract	5	5 (100)	4 (80)	4 (80)	1 (20)	1 (20)	3 (60)	5	5 (100)
23	Psychotherapeutic medicines	7	7 (100)	6 (86)	4 (57)	1 (14)	2 (29)	4 (57)	13	10 (77)
24	Medicines acting on the respiratory tract	10	10 (100)	8 (80)	6 (60)	2 (20)	9 (90)	7 (70)	10	9 (90)
25	Solutions correcting water, electrolytes and acid–base disturbances and parenteral nutrition	7	6 (86)	7 (100)	6 (86)	3 (43)	4 (57)	5.2 (74)	10	10 (100)
26	Vitamins and minerals	2	2 (100)	1 (50)	1 (50)	1 (50)	1 (50)	1.2 (60)	4	2 (50)
27	Medicines acting on the ear, nose and oropharynx	9	5 (56)	2 (22)	2 (22)	3 (33)	3 (33)	3 (33)	9	4 (44)
28	Specific medicines for neonatal care	1	1 (100)	0	0	0	0	0.2 (20)	2	2 (100)
	Total (%)	165	134 (81)	105 (64)	96 (58)	52 (32)	78 (47)	112.6 (56)	251	179 (71)

Duplication of medicines, in multiple categories of the national list of essential medicines, Sri Lanka (2013–2014), was left out of the calculation of availability (%). N/A - These categories of the national list of essential medicines, Sri Lanka (2013–2014), are not applicable for primary care institutions. *PCI* Primary care institution, *SCI* Secondary care institution, *BH* Base Hospital, *DH* District Hospital, *PU* Peripheral Unit, *RH* Rural Hospital, *CD* Central Dispensary

PU showed 100% availability in diuretics and solutions correcting electrolytes. “Low” or “very low” availability at PU was recorded with anticonvulsants (46%), medicines affecting the blood (33%), dermatological preparations (44%), diagnostic agents (0%), immunologicals (0%), ophthalmological preparations (0%), ENT medicines (22%) and medicines for neonatal care (0%) (Table 2).

RH showed 100% availability in diuretics only, whereas both the CDs failed to show 100% availability in any of the major categories of essential medicines (Table 2). Diagnostic agent (tropicamide eye drops) was not available at any of the primary care institutions. Immunological (anti-venom serum) and medicines for neonatal care (chlorhexidine for umbillical cord) were only available at DH. Anticonvulsants were not available in both the CDs, whereas CD-2 failed to have any of the antidotes or other substances used for poisoning (Table 2).

### Secondary care institution

BH, the only secondary care institution of the survey, had an overall availability of 71%. It had shown 100% availability in anaesthetics, anti-allergics, blood products, antiseptics, diuretics, immunologicals, muscle relaxants, genitourinary medicines, solutions correcting electrolytes and medicines for neonatal care. “Low” or “very low” availability was seen in anti-parkinsonism medicines (25%), dermatological preparations (33%), diagnostic agents (0%), ophthalmological preparations (0%) and ENT medicines (44%) (Table 2).

### Comparison of availability of essential medicines between primary and secondary care institutions

BH has a higher overall availability (71%) than the average overall availability of all primary care institutions (56%). BH also had most number of drugs (179 vs DH - 134, PU - 105, RH - 96, CD-1 - 52, CD-2 - 78). Secondary care institution showed higher availability in 18 major categories compared to the primary care institutions. However, secondary care institution has recorded lower availability in analgesics, anti-infectives, dermatological preparations, ophthalmological preparations and vitamins and minerals (Table 2).

**Fig. 1:**
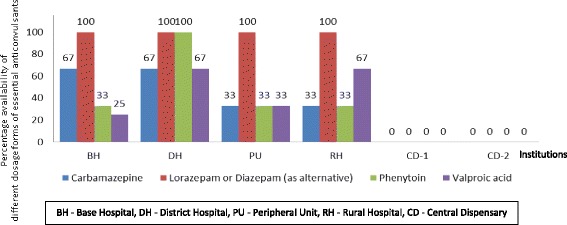
Percentage availability of different dosage forms of essential anticonvulsants, Anuradhapura, 2016

**Fig. 2:**
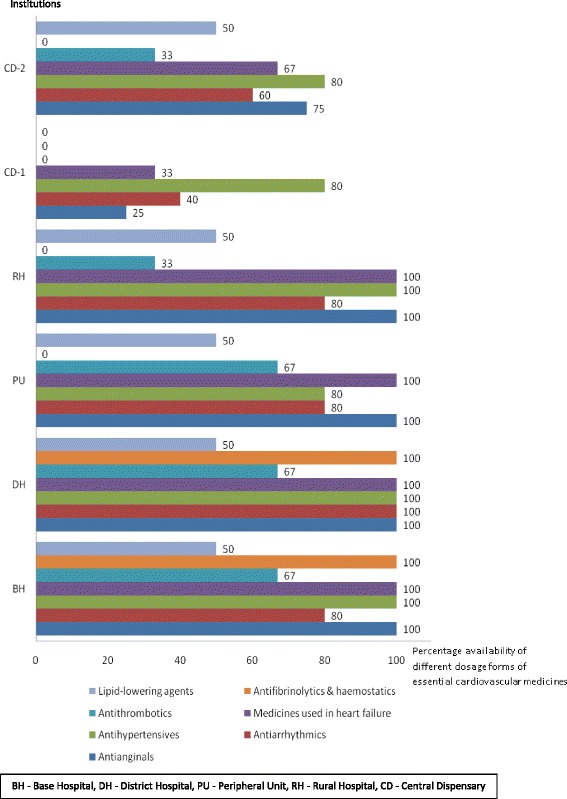
Percentage availability of different dosage forms of essential cardiovascular medicines, Anuradhapura, 2016

The secondary care institution failed to have any ophthalmological preparations but 4 out of the 5 primary care institutions (DH, RH, CD - 1, CD - 2) have had ciprofloxacin eye drops. Medicines acting in nose (betamethasone nasal drop, silver nitrate crystals) were not found in any of the institutions selected for the study. Out of all institutions only DH had povidone iodine mouth-wash. Aqueous cream and emulsifying ointment were found only in DH, even the secondary care institution failed to have it. Availability of anticonvulsants was 50% at the secondary care institution compared to the mean availability of 36% at the primary care institutions (Fig. 1). Availability of cardiovascular medicines was 89% at the secondary care institution compared to the mean availability of 70% at the primary care institutions (Fig. 2).

### Availability of medicines used in the management of chronic kidney disease

Essential medicines involved in the management of chronic kidney disease were ferrous salt, iron sucrose, erythropoietin, vitamin D3 + calcium carbonate and calcitriol [19].

Erythropoietin and calcitriol are not expected to be available in primary or secondary care institutions. Rest was expected to be available at all levels of care. The secondary care institution (BH) failed to have ferrous sulphate tablets used in the management of iron deficiency anaemia. In comparison 4 out of 5 primary care institutions (DH, PU, RH, CD-2) have had ferrous sulphate tablets. Intravenous iron preparation (iron sucrose) was not found in any of the institutions. Vitamin D3 + calcium carbonate was found only in DH.

Anti-hypertensive medicines expected to be available at primary and secondary care levels were atenolol, enalapril, hydrochlorothiazide, methyldopa and nifedipine. Except for hydrochlorothiazide and methyldopa, all other are used in chronic kidney disease. Secondary care institution had all of them. Methyldopa, used to treat pregnancy induced hypertension was not available in PU, CD-1 and CD-2. However, all other anti-hypertensive medicines were found in all primary care institutions.

Furosemide, hydrochlorothiazide, mannitol and spironolactone are the diuretics expected to be available at a secondary care institution. BH had all of them. Out of the diuretics mentioned above, mannitol is not expected to be available at primary care institutions. DH, PU and RH showed 100% availability for diuretics. CD-1 had only hydrochlorothiazide. CD-2 had all except intravenous furosemide.

### Insulin and other anti-diabetic agents

Biphasic insulin, metformin, glibenclamide and tolbutamide were expected to be available in primary care units. DH, PU and RH had all of those drugs. CD-1 had metformin and glibenclamide. CD-2 in addition had tolbutamide. Soluble insulin is expected to be present in secondary care institutions in addition to the above medicines. BH had all of them.

### Drugs needed in the management of snake bite

Snake anti-venom serum injection was available only at BH and DH. Adrenaline, hydrocortisone and parenteral chlorphenamine, which are essential in the management of anaphylaxis following anti-venom serum administration, were available in all institutions surveyed.

### Antidotes (Table 3)

**Table 3:** Availability of essential antidotes at selected primary and secondary health care institutions of Anuradhapura, 2016

Antidote	Institutions					
	BH	DH	PU	RH	CD-1	CD-2
Non-specific antidotes						
Charcoal (Activated)	P	P	P	P	A	A
Fuller’s earth	A	P	P	A	A	A
Specific antidotes						
Acetylcysteine	P	NA	NA	NA	NA	NA
Atropine	P	P	P	P	P	A
Flumazenil	P	NA	NA	NA	NA	NA
DL-methionine	P	A	A	A	A	A
Naloxone	P	A	A	A	A	A
Pralidoxime	P	NA	NA	NA	NA	NA

*BH* Base Hospital, *DH* District Hospital, *PU* Peripheral Unit, *RH* Rural Hospital, *CD* Central Dispensary, *P* Present, *A* Absent, *NA* Not applicable

Secondary care institution is expected to have activated charcoal, fuller’s earth, acetylcysteine, atropine, flumazenil, DL-methionine, naloxone and pralidoxime; but fuller’s earth was not available. Activated charcoal, fuller’s earth, atropine, DL-methionine and naloxone were expected to be available in primary care units. Both CDs failed to have any of them, except for atropine at CD-1. PU, RH and DH had activated charcoal and atropine. These two are essential in the management of organophosphate poisoning [20], the commonest poisoning of the agricultural areas in SL.

## Discussion

According to the WHO definition, essential medicines are expected to be available at all times within a functioning health system [1]. Our study mimicked a patient visiting a selected institution with a need of a particular essential medicine. This gives an insight to the availability of essential medicines in those selected institutions.

DH is the only health care institution to have a “high” availability of essential medicine. BH, PU and RH had “fairly high” availability, whereas both the CDs had “low” availability. A national survey done in SL revealed an availability of 36.4% and >50% respectively for primary and secondary levels of care. The above survey was on selected essential medicines used in non-communicable diseases [6]. Another national survey revealed 54, 49 and 45% as the mean availability of key essential medicines for children in district hospitals, peripheral hospitals and central dispensaries of SL [7].

Specialist units like ophthalmology, otorhinolaryngology, dermatology and neonatology are not available in primary or secondary care levels. Also number of deliveries is low in primary care institutions. These reasons could have contributed to the unavailability of essential medicines related to these specialities.

More than 70% of availability in BH and DH reflects larger number of patients utilizing services of these institutions. Although BH (a secondary care institution) has lower availability of analgesics and anti-infectives it is a relative unavailability compared to some primary care institutions and the essential list. Eight out of 12 analgesics and 32 out of 47 anti-infectives are available in BH compared to 6/6 and 20/24 in DH. Careful analysis of data (Additional file 1) displays that only some dosage forms of anti-infectives are not available in BH. Only the infrequently needed co-trimoxazole and clindamycin are not available in any form or strength. Among analgesics, only morphine sulphate tablets are not available in BH. This may reflect lack of facility for palliative care. Availability of morphine sulphate tablets in the public sector was 4% in SL as reported by a study in 2007 [4].

Anuradhapura district is an endemic area for chronic kidney disease. Lack of availability of medicines related to chronic kidney disease was noted. Also a national list of essential medicine cannot address regional variation of pharmaceutical need and utilization. Certain medicines should be available in all levels of health care for some parts of the country. Tailoring the essential medicines to suit the needs of a particular region is suggested. Unavailability of intravenous iron even in a base hospital may suggest genuine unavailability or lack of utilization. Intravenous iron is most suitable for treatment of iron deficiency in chronic kidney disease and rapid treatment in de novo iron deficiency. Overall, Availability of medicines needed for the management of hypertension and diabetes was commendable. Availability of anti-diabetics from a previous national survey in SL was nearly 60% in public hospitals [6]. The same survey revealed an availability ranging from 37 to 100% for anti-hypertensive medicines.

Snake anti-venom serum injection was available only at DH among the primary care institutions. Considering the higher prevalence of venomous snake bites in SL dry zones [21] this situation needs to be rectified immediately. Higher mortality ratescan be reduced to a certain extent by preventing unnecessary delay in starting the anti-venom serum. This situation is despite the fact that all institutions had medicines needed for managing anaphylactic shock following anti-venom serum administration. However the efficacy of Indian anti-venom serum (VINS and BHARAT are used in SL) against SL snake venom is questionable [22].

Atropine, the antidote for organophosphate was available in all health care institutions except CD-2. A study conducted in Anuradhapura has shown that pesticides were the commonest (1572/3813 - 41% of total admissions due to poisoning) agents for poisoning followed by pharmaceuticals (21% of total admissions due to poisoning) [23]. However the same study also shows that the pattern of deliberate self-harm is changing with increased use of pharmaceuticals. This mandates availability of wider range of antidotes. Unavailability of fuller’s earth in most institutions may be due to the banning of the specific poisoning “paraquat” since 2010.

Both the CDs have shown unavailability of certain anticonvulsants and antidotes which are needed for the management of medical emergencies (Table 3, Fig. 1). The concept of having four levels of care encourages initial management and stabilization of an acutely ill patient at the first contact. Uninterrupted supply of essential medicines used in emergencies is needed to achieve this goal.

Chlorhexidine solution for umbillical cord care was available only at DH among the primary care institutions. According to WHO recommendations “daily chlorhexidine (7.1% chlorhexidine digluconate aqueous solution or gel, delivering 4% chlorhexidine) application to the umbilical cord stump during the first week of life is recommended for newborns who are born at home in settings with high neonatal mortality (30 or more neonatal deaths per 1000 live births). Clean, dry cord care is recommended for newborns born in health facilities and at home in low neonatal mortality settings. Use of chlorhexidine in these situations may be considered only to replace application of a harmful traditional substance, such as cow dung, to the cord stump” [24]. Neonatal mortality in Anuradhapura is 24 neonatal deaths per 1000 live births according to the latest available data [25]. Institutional delivery rate in SL is more than 98% [26]. The above facts justify the non-usage of chlorhexidine and may have been the reason for unavailability. Also when there are no deliveries or neonatal care in certain hospitals (CD-1 and CD-2), medicines related to pregnancy (such as methyldopa in pregnancy induced hypertension) and neonatal care (such as chlorhexidine in umbilical cord care) may not be needed.

Tropicamide eye drops are not available in any of the surveyed institutions. They are being used to dilate the pupils for examination of retina [27]. Retinopathy survey in diabetes seems to be absent in any of the surveyed institutions.

The national list of essential medicines was written exclusively by experts based at Colombo, an urban capital district of SL. “Experts” may not be available from the rural areas. However, inputs from prescribers working in rural areas may enhance the list of essential medicines and make it more relevant for prescribers in rural areas. Also having a separate list of essential medicines, for primary care institutions which do not have inward facilities, is suggested.

The concept of “essential medicines” is yet to be followed by the individual heads of health care institutions. Unavailability of certain medicines may be due to non-utilization of those medicines by prescribers. Medicines are supplied purely on the annual estimates prepared by the relevant institutions based on usage in the previous year. This would predict the quantity needed for the following year but will fail in making available all varieties of the essential medicines. Continuous medical education is needed to enlighten prescribing doctors on the importance of utilizing medicines available in the national list of essential medicines.

Regardless of all these short coming SL has achieved health indices comparable to developed countries [8] and many nationwide studies have also shown a “fairly high” availability of essential medicines in SL [4, 6] which was supported by this survey too.

## Conclusions

Overall availability of essential medicines at the selected institutions was fairly high. It was high at BH, a primary care institution, and low at both the CDs. There is concern in the availability of antidotes, snake anti-venom and medicines used in the management of chronic kidney disease. However availability of anti-hypertensive medicines, anti-diabetics and diuretics was commendable yet there is room for improvement. This will reduce not only the overcrowding at tertiary care institutions but also the indirect health care cost for rural residents. Amalgamating the concept based “essential medicines” with the need based “annual estimates” is suggested for improving the availability of essential medicines.

## Additional file

Additional file 1: Availability of essential medicines in a rural Sri Lankan district, 2016. This provides the results of the entire survey with data for each essential medicine. (XLS 187 kb)

## Abbreviations

BH: Base Hospital
CD: Central Dispensary
DH: District Hospital
HAI: Health Action International
PU: Peripheral Unit
RH: Rural Hospital
SL: Sri Lanka
WHO: World Health Organization
